# Supplementary material for: The Effects of Sequence Length and Composition of Random Sequence Peptides on the Growth of E. coli Cells
Source: Genes (Basel). 2021 Nov 28;12(12):1913. doi: 10.3390/genes12121913 (PMC8702183; doi:10.3390/genes12121913)
Supplement: Supplementary file 1 [file genes-12-01913-s001.zip › genes-1445543-supplementary.pdf]

The Effects of Sequence Length and Composition of Random Sequence Peptides on the Growth of *E. coli* Cells

Johana Fajardo, and Diethard Tautz#

Supplementary files

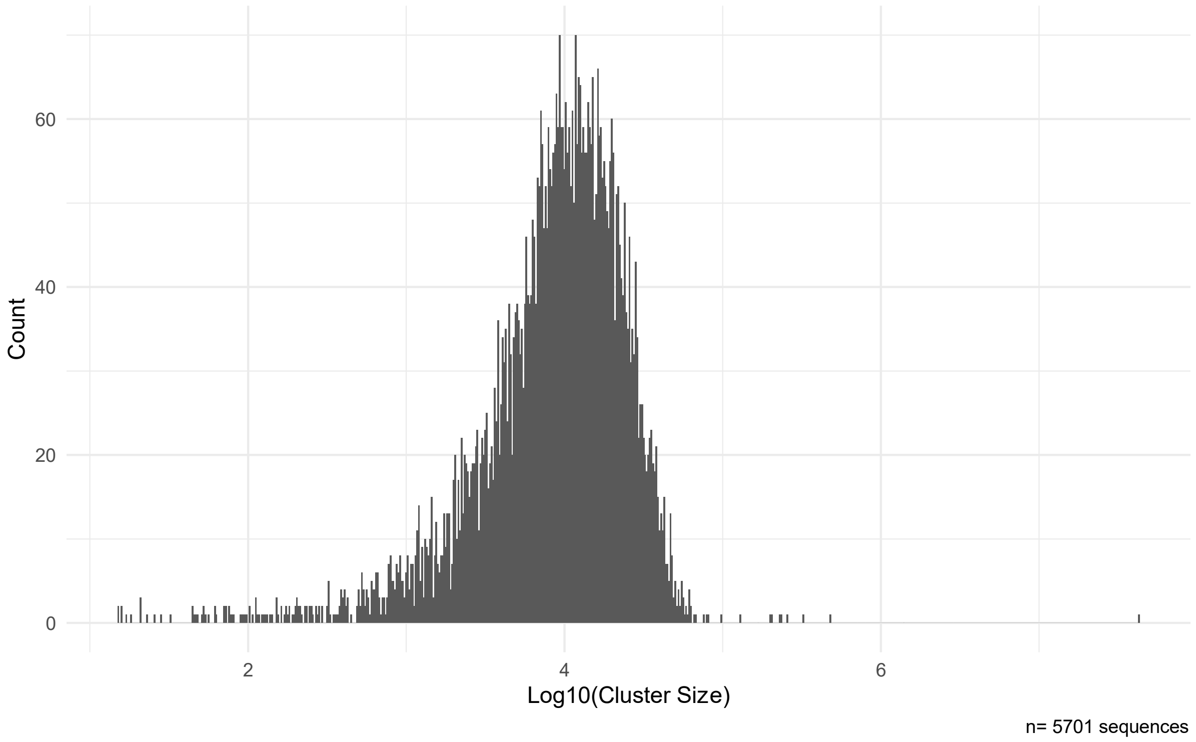

Figure S1
Cluster size distribution of clones. Cluster sizes correspond to the number of reads across all experiments assigned to each of the 5,701 clones in the database. Most clones had between 103 and 105 reads assigned to them (mean 2.03x104 reads, median 9.9x103 reads). There is a single clone with 4.23x107 reads, which corresponds to the pFLAG-CTC plasmid without an insert.

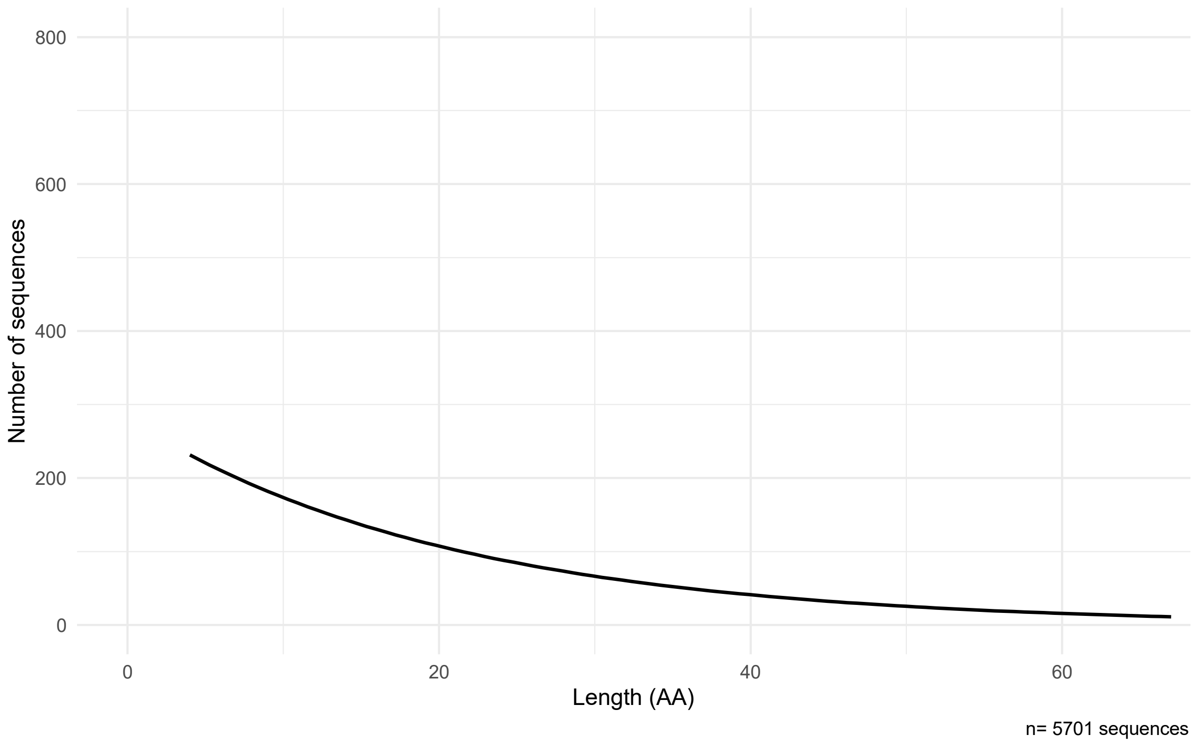

Figure S2
Predicted peptide length distribution of all clones in the library. The library follows the expected distribution of stop codons in 5,701 random sequences, defined as the probability mass function of a geometric distribution with p = 3/64 stop codons (black line). Only the first ORF in each sequence was considered. Minimum peptide length corresponds to the 4 residues encoded by the plasmid and insert design (MKLS). Residues 55 to 65 are constant in the sequence design (ALVDYKDDDDK*), which should result in a single peak for sequences of length 65. However, the process of library synthesis or cloning generated 1735 clones with unexpected sequence lengths (30.43% of the clones in the library). This resulted in 171 clones with predicted peptide lengths between 55 and 64 residues or longer than 65 residues.

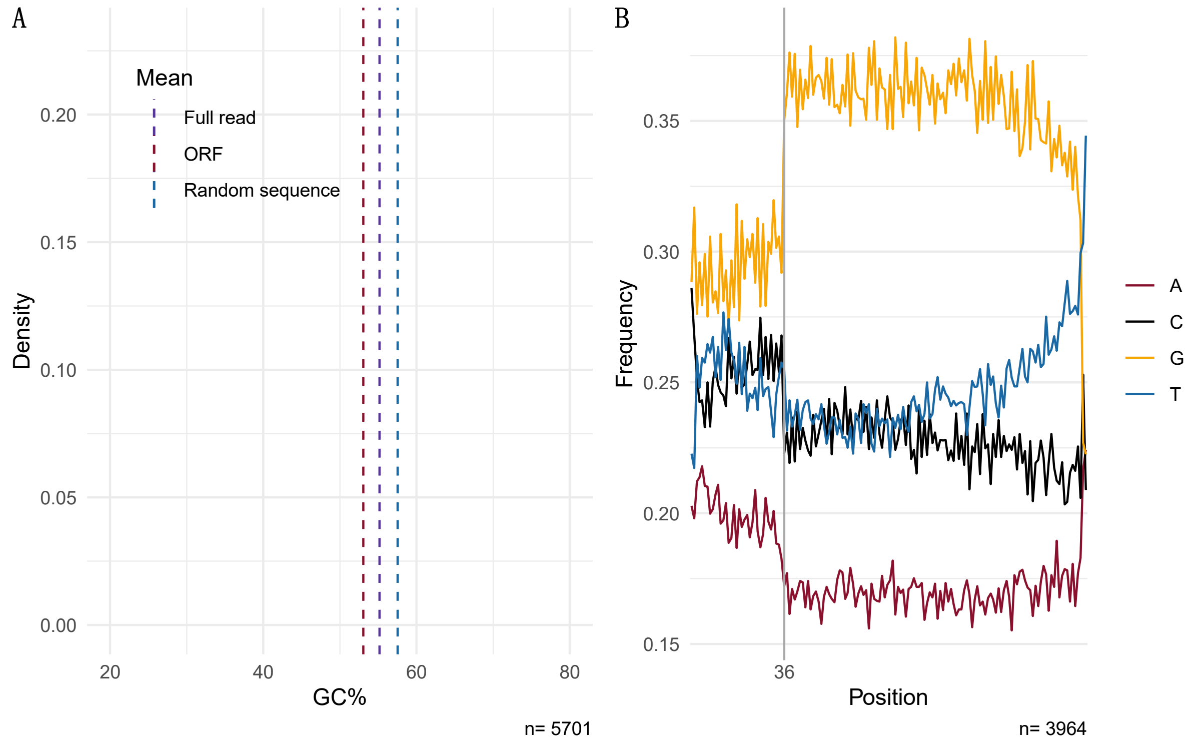

Figure S3
GC content analysis of clones in the library.
A) GC content distributions calculated over the complete reads, between start and FLAG-tag sequences (this includes the flanking sequences of the plasmid, with a GC content of 46.2%the full sequenced reads (purple), the ORFs only (red) and simulated random sequences (blue). The ORF GC distribution is much more broadly spread, due to increased variance caused by very short ORFs.
B) Fraction of each nucleotide along the positions of the randomly synthesized sequence stretch.

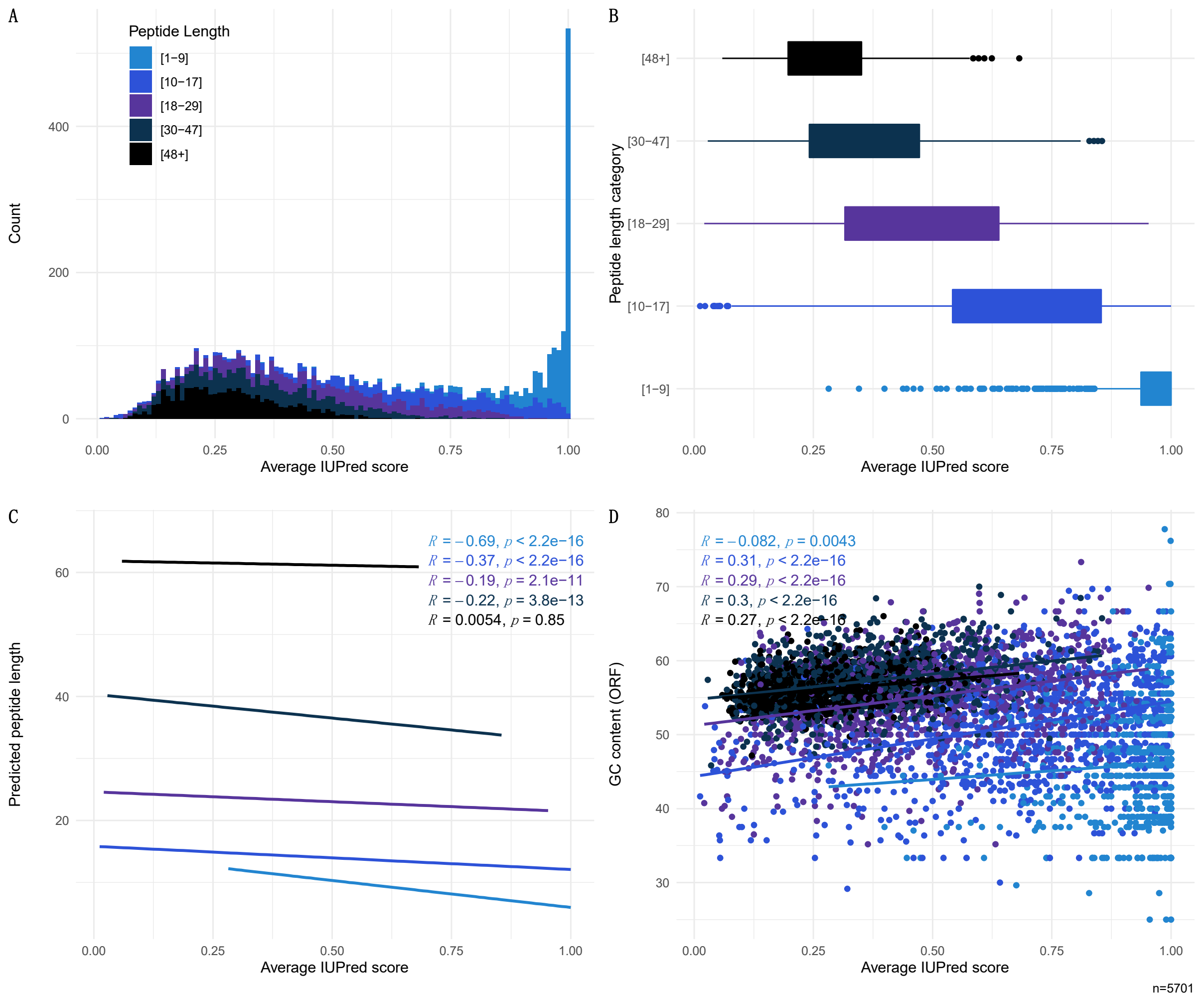

Figure S4

Intrinsic disorder scores for peptides in the whole library in four different representations (A-D; see main text). Average intrinsic disorder scores become smaller for longer peptides (length 1-9 = 0.947, length 10-17 = 0.677, length 18-29 = 0.479, length 30-47 = 0.362, length 48+ = 0.281.

Table S1: List of primers used

pFLAG-CTC FWD-1	AATGATACGGCGACCACCGAGATCTACAC AACCGCAT ACACTCTTTCCCTACACGACGCTCTTCCGATCT CATCATAACGGTTCTGGCAAATATTC
pFLAG-CTC FWD-2	AATGATACGGCGACCACCGAGATCTACAC AAGGCCTT ACACTCTTTCCCTACACGACGCTCTTCCGATCT T CATCATAACGGTTCTGGCAAATATTC
pFLAG-CTC FWD-3	AATGATACGGCGACCACCGAGATCTACAC AGAGTGTG ACACTCTTTCCCTACACGACGCTCTTCCGATCT GT CATCATAACGGTTCTGGCAAATATTC
pFLAG-CTC FWD-4	AATGATACGGCGACCACCGAGATCTACAC CACAAGTC ACACTCTTTCCCTACACGACGCTCTTCCGATCT CGA CATCATAACGGTTCTGGCAAATATTC
pFLAG-CTC FWD-5	AATGATACGGCGACCACCGAGATCTACAC CGTTCCTA ACACTCTTTCCCTACACGACGCTCTTCCGATCT ATGA CATCATAACGGTTCTGGCAAATATTC
pFLAG-CTC FWD-6	AATGATACGGCGACCACCGAGATCTACAC GCTTGGAT ACACTCTTTCCCTACACGACGCTCTTCCGATCT TGCGA CATCATAACGGTTCTGGCAAATATTC
pFLAG-CTC FWD-7	AATGATACGGCGACCACCGAGATCTACAC GTCAACAC ACACTCTTTCCCTACACGACGCTCTTCCGATCT GAGTGG CATCATAACGGTTCTGGCAAATATTC
pFLAG-CTC RWD-A	CAAGCAGAAGACGGCATACGAGAT AACCGGAA GTGACTGGAGTTCAGACGTGTGCTCTTCCGATCT A CTGTATCAGGCTGAAAATCTTCT
pFLAG-CTC RWD-B	CAAGCAGAAGACGGCATACGAGAT AGAGTGAC GTGACTGGAGTTCAGACGTGTGCTCTTCCGATCT TC CTGTATCAGGCTGAAAATCTTCT
pFLAG-CTC RWD-C	CAAGCAGAAGACGGCATACGAGAT CAACTGGT GTGACTGGAGTTCAGACGTGTGCTCTTCCGATCT CTA CTGTATCAGGCTGAAAATCTTCT
pFLAG-CTC RWD-D	CAAGCAGAAGACGGCATACGAGAT CGTTCGTT GTGACTGGAGTTCAGACGTGTGCTCTTCCGATCT GATA CTGTATCAGGCTGAAAATCTTCT
pFLAG-CTC RWD-E	CAAGCAGAAGACGGCATACGAGAT CTGTTCAC GTGACTGGAGTTCAGACGTGTGCTCTTCCGATCT ACTCA CTGTATCAGGCTGAAAATCTTCT
pFLAG-CTC RWD-F	CAAGCAGAAGACGGCATACGAGAT GCTTGCAA GTGACTGGAGTTCAGACGTGTGCTCTTCCGATCT TTCTCT CTGTATCAGGCTGAAAATCTTCT
pFLAG-CTC RWD-G	CAAGCAGAAGACGGCATACGAGAT GTCAACTG GTGACTGGAGTTCAGACGTGTGCTCTTCCGATCT CACTTCT CTGTATCAGGCTGAAAATCTTCT

Table S2: Amino acid frequencies for the database, as well as the three groups of peptides

AA	Database	UP	DOWN	NS
W	0.0284	0.0279	0.0298	0.0268
F	0.0320	0.0345	0.0326	0.0304
Y	0.0221	0.0204	0.0221	0.0224
I	0.0314	0.0294	0.0325	0.0306
M	0.0156	0.0134	0.0164	0.0153
L	0.0918	0.0957	0.0915	0.0913

V	0.0850	0.0816	0.0878	0.0825
N	0.0189	0.0200	0.0191	0.0185
C	0.0431	0.0462	0.0441	0.0414
T	0.0448	0.0422	0.0450	0.0437
A	0.0884	0.0963	0.0869	0.0895
G	0.1134	0.1034	0.1162	0.1136
R	0.1141	0.1116	0.1164	0.1118
D	0.0268	0.0257	0.0257	0.0277
H	0.0243	0.0253	0.0235	0.0251
Q	0.0264	0.0280	0.0250	0.0277
K	0.0205	0.0190	0.0204	0.0201
S	0.0904	0.0974	0.0852	0.0951
E	0.0282	0.0264	0.0282	0.0286
P	0.0545	0.0558	0.0516	0.0580
